# Coupling of Anodic Stripping Voltammetry with Sampled-Current Voltammetry on an Electrode Array: Application to Lead Detection

**DOI:** 10.3390/s20051327

**Published:** 2020-02-29

**Authors:** Isabelle Mazerie, Florence Geneste

**Affiliations:** Univ Rennes, CNRS, ISCR-UMR 6226, F-35000 Rennes, France; i.mazerie@gmail.com

**Keywords:** electrochemical sensor, electrode array, sampled-current voltammetry, lead, anodic stripping voltammetry

## Abstract

Electrochemical detection systems are very promising for pollution monitoring owing to their easy miniaturization and low cost. For this purpose, we have recently developed a new concept of device based on Electrodes Array for Sampled-Current Voltammetry (EASCV), which is compatible with miniaturization and portability. In this work, to improve the sensitivity of the analytical method, we added a preconcentration step before EASCV analysis, combining sampled-current voltammetry with anodic stripping voltammetry. Lead was chosen as analyte for this probe of concept owing to its high toxicity. The conditions for electrodeposition of lead on gold were optimized by means of under potential deposition. Current intensities 300 times higher than with linear sweep anodic stripping voltammetry were obtained, showing the interest in the method. The value of the sampling time directly affected the sensitivity of the sensor given by the slope of the linear calibration curve. The sensor exhibited a limit of detection of 1.16 mg L^−1^, similar to those obtained with linear sweep anodic stripping voltammetry.

## 1. Introduction

Heavy metals such as lead, mercury and cadmium are very toxic even at trace level with European drinking water guidelines set at 10, 1 and 5 μg/L, respectively [1]. Owing to their omnipresence in the ecosystem, they represent a high risk for human health and environment [2]. To facilitate site monitoring, a portable analytical system for heavy metal detection at trace level allowing a fast analysis is required. However, the preparation of such an analytical system is still challenging. Electroanalysis is one of the most promising methods to meet this need [3,4,5,6,7,8]. Research on trace heavy metal analysis with portable systems mainly focus on anodic stripping voltammetry that allows the detection of very low concentrations of metallic ions by their preconcentration on the electrode surface. The use of hanging mercury electrodes allows high reproducibility and sensitivity due to a high affinity between mercury and the reduced metallic cations leading to the formation of amalgams Hg (metal) [9]. This phenomenon facilitates the preconcentration of the metal at the electrode surface. However, the interest in achieving a portable device considering the environmental issues leads to the development of new analytical systems without mercury. Gold electrodes have been proposed as an alternative and have been used for the detection of heavy metals such as copper, mercury and lead [10,11,12,13,14,15]. The preparation of screen-printed sensors or made by photolithography with a bare or modified gold electrode has been also reported for lead detection [12,16,17].

Electrochemical studies on this field focused on more sensitive, reproducible and selective systems. The improvement of the detection of heavy metals at trace level requires the optimization of anodic stripping voltammetry. Thus, Krowa-Eisner et al. used Subtractive Anodic stripping voltammetry (SASV) [18,19]. This technique consists in linear sweep anodic stripping voltammetry followed by a linear voltammetry analysis without deposition. The data were treated by subtraction of the two resulting curves [10,11,18,19,20,21]. Although effective, this technique requires rather complex informatics data processing for a portable application. Anodic stripping voltammetry has also been optimized with Under Potential Deposition (UPD). It consists in an electrodeposition step performed at a potential less negative than the equilibrium of the studied species [21], avoiding the formation of multilayers. It simplifies the electrochemical signal avoiding the presence of several peaks after oxidation.

We have previously reported a new analytical method called Electrodes Array for Sampled-Current Voltammetry (EASCV) based on sampled-current voltammetry performed on an electrode array [22,23]. A potential was applied independently on each electrode of the array and the resulting current was read at a short sampling time. A current-potential curve was therefore obtained by plotting the current *versus* the applied potential. Thus, the renewal of the electrode surface was assured and a fresh solution was always available close to the electrode surface. Since the data acquisition that does not require the use of a potential ramp was simplified, the device can be portable and cost-effective. Our previous studies focused on the interest in the method to mimic dropping mercury electrodes and to circumvent the problem of passivation during analysis.

The aim of this new study is to combine an electrodeposition step with EASCV for heavy metal detection. The use of an electrode array in EASCV allows a coupling of methods that was not possible before with dropping mercury electrodes. Since a new electrode covered by the same amount of metal will be analyzed at each applied potential, high current intensities will be expected with a simplified data processing for an easier adaptation to portable device. Lead detection was chosen in this work as an example of application.

## 2. Experimental Part

### 2.1. Reagents and Materials

Lead (II) nitrate or Lead (II) chloride 99% were purchased from Acros and Aldrich, France, respectively. All solutions were prepared with ultra-pure water (18.2MX, Millipore Simplicity, France). All glassware and the electrochemical cell were rinsed with a 10% HNO_3_ solution followed by ultra-pure water before use to avoid metal contamination.

### 2.2. Preparation of the Electrodes

The electrodes prepared by photolithography [22,24] were cleaned in acetone with slow stirring (50 rpm) for 5 min and rinsed with ultra-pure water. After drying under vacuum, the electrode array was treated for 25 min with UV/ozone. Then it was dipped twice in acetone, acetonitrile and ethanol for 5 min, and rinsed abundantly with ultra-pure water before being dried with argon. Finally, before electrochemical analyses, all the working electrodes were electrically connected and cyclic voltammetry was performed between 1.5 and −1 V/SCE until a stable electrochemical signal was obtained in phosphate buffer at neutral pH.

After analysis, the electrodes were regenerated in HNO_3_ 69%. After rinsing abundantly with ultra-pure water, the electrode array was washed in several baths of ultra-pure water with slow stirring (50 rpm).

### 2.3. Electrochemical Analysis

The electrochemical analyses were performed in a standard three-electrode configuration, with a platinum wire counter electrode, a saturated calomel reference electrode (SCE) and a gold electrode depending on the experiment:

(1) A gold bar (∅ = 3 mm, geometric area 0.07 cm^2^) inserted in a tube of glass (polished before each experimentation)

(2) An electrode array (∅ = 0.5 mm) made with photolithography process [22].

All the electrochemical experiments were performed at room temperature (25 °C) under deaerated conditions in an electrochemical cell adapted to the electrode (for a classical single electrode the cell contains 20 mL of liquid)

**Figure 1:**
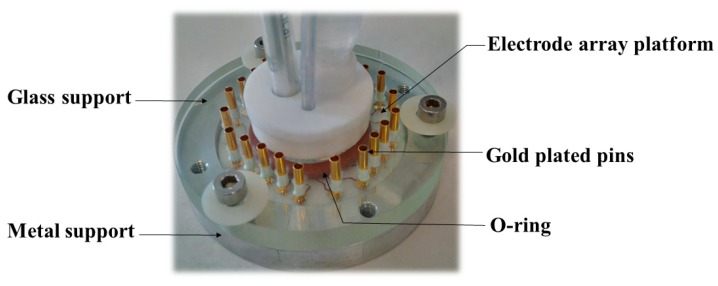
Electrochemical cell used for Electrodes Array for Sampled-Current Voltammetry (EASCV) analysis.

Concerning the homemade electrode array the electrochemical experiments were performed in a homemade electrochemical cell (Figure 1) [22]. The first piece in metal supports the electrode array platform and a second one made of glass contains 22 sprung gold plated pins, which assure electrical contacts and a 15 mm diameter tank for the reception of liquids and electrodes. The geometric area of each electrode of the array was 0.002 cm². The cylindrical vessel was sealed with an o-ring that was clamped between the top and bottom plates. In total, 3 mL of solution were used.

Cyclic voltammetry and chronoamperometry experiments were carried out with a VersaSTAT3 AMETEK^®^ Model potentiostat/galvanostat with a versaSTAT LC Low Current Interface (Princeton Applied Research) and the versaStudio Software.

The electrochemical analysis was performed in ultra-pure water, containing either 0.1 mol L^−1^ sodium chloride or 0.01 mol L^−1^ KNO_3_ potassium nitrate as supporting electrolyte according to the nature of the lead salts.

## 3. Results and Discussion

### 3.1. Optimization of the Electrochemical Signal

#### 3.1.1. Cyclic Voltammetry Analysis

**Figure 2:**
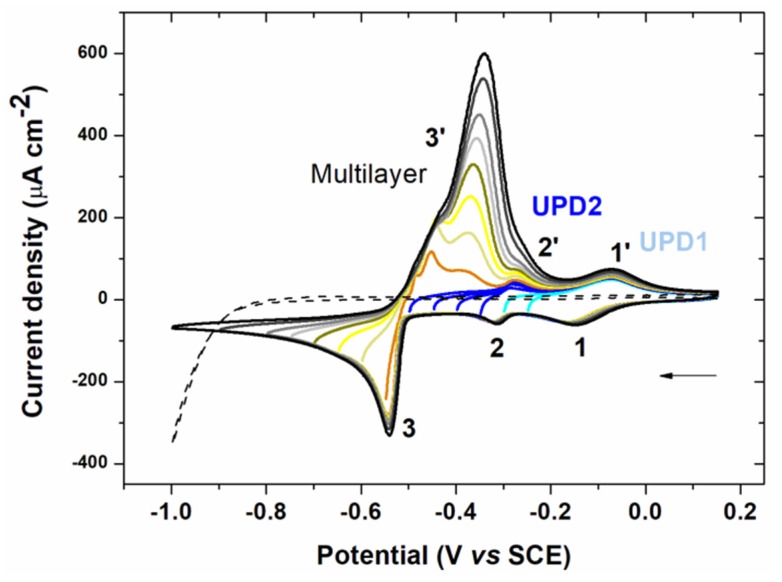
Voltammograms of a 10^−3^ mol L^−1^ solution of lead(II) chloride in 0.1 mol L^−1^ NaCl on gold electrode (0.07 cm²) with decreasing negative potential limits and blank (-----). The solution was degassed for 5 min between each cycle. Scan rate: 100 mV s^−1^.

The electrochemical signal of lead depends on the nature of the electrode. In this study, gold was chosen as a good alternative to mercury [12,13,14] and owing to the nature of the electrode array that will be used in EASCV analysis [22]. Cyclic voltammetry analysis of lead performed on a gold electrode is given in Figure 2. The electrode was polished between each analysis, changing the negative potential limit.

Two cathodic peaks beginning at −0.025 V/SCE (1) for UPD1 and −0.275 V/SCE (2) for UPD2 appeared at more positive potentials than the lead equilibrium potential (−0.46 V/SCE). This phenomenon called UPD occurs when a metal is deposited on an electrode of different nature. Thus, the metal is deposited at a potential higher than its equilibrium potential due to a difference in affinity between the metal and the surface of the electrode compared with the metal-metal interaction. The interactions between the metal and the electrode surface are stronger than the metal-metal interactions. Therefore the energy required to reduce the first layer of metal is less important than that required for multilayer formation [21]. The appearance of two UPD systems in Figure 2 can be explained by the differences in the electrode structure (e.g., poly or monocrystalline gold) [25,26,27,28,29]. Then the deposition of the multilayer metal started at −0.5 V/SCE (3) after the equilibrium potential. This multilayer deposition defined as overpotential deposition had a typical shape of a phase change (abrupt decrease in the current). The first oxidation peaks (3’) corresponded to the dissolution of lead deposited in multilayer. A double peak was visible, which was probably a consequence of the lateral interactions between the atoms [30]. Then two anodic peaks corresponding to UPD2 (2’) and UPD1 (1’) appeared.

The UPD signal in stripping voltammetry analysis offers several advantages [21]. First, for trace analysis, only a small part of the working electrode is covered during deposition. Thus the presence of the peak corresponding to UPD is assured unlike the main oxidation peak after multilayer deposition. Second, focusing on a well-defined peak provides improved reproducibility. Finally, since only one layer is deposited, the preconcentration step requires a shorter time, reducing the analysis time and is made at a less cathodic deposition potential. This last point allows the decrease in the potential window for the analysis giving access to electrodes with low hydrogen overvoltage such as gold. For these reasons, stripping voltammetry analysis was focused on the peaks corresponding to UPD in the following experiments.

#### 3.1.2. Electrodeposition Potential

**Figure 3:**
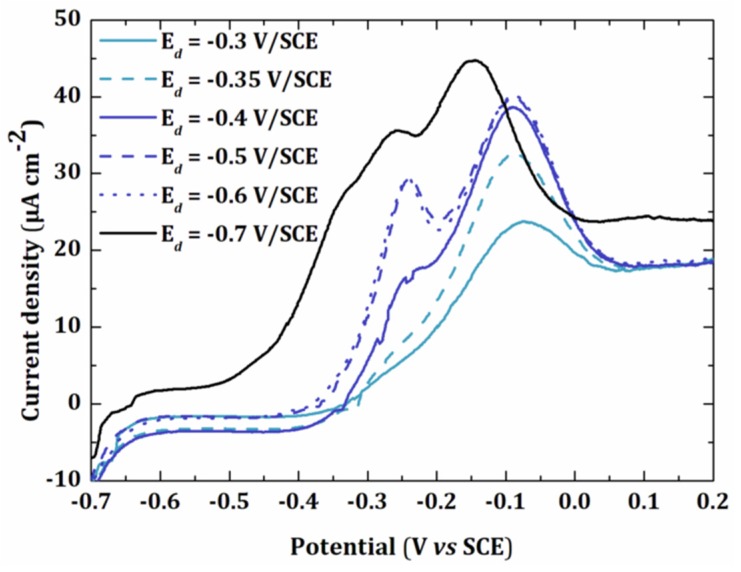
Linear sweep voltammetry of a 5 × 10^−6^ mol L^−1^ solution of Pb(NO_3_)_2_ in 0.01 mol L^−1^ KNO_3_ at gold electrode (0.002 cm²) for a deposition time of 60 s at different deposition potentials (−) UPD1, (−)UPD1 + UPD2, (−) UPD1 + UPD2 + multilayer.

Different electrodeposition potentials (Ed) were tested ranging from −0.3 V/SCE to −0.7 V/SCE for the same deposition time (td = 60 s) (Figure 3).

The peak current increased when the electrodeposition potential was more negative. A first peak was observed at −0.075 V/SCE. The presence of a shoulder at −0.25 V/SCE for Ed = −0.35 V/SCE showed the appearance of a second peak, which was clearly visible when Ed = −0.5 V/SCE. Finally, a third peak appeared for Ed = −0.7 V/SCE. This peak probably corresponded to the multilayer deposition of lead since it occurred at −0.53 V/SCE under these conditions (Figure 2). It has been shown that each new peak only appears when the first peak has reached its maximum intensity, even though the potential is negative enough for the formation of several peaks [29]. In this work, to avoid multilayer deposition of lead, we used a deposition potential of −0.5 V/SCE.

#### 3.1.3. Electrodeposition Time

To study the effect of the electrodeposition time, a solution of 5 × 10^−7^ mol L^−1^ lead chloride was electrodeposited at a potential of −0.5 V/SCE on a gold electrode of the array.

**Figure 4:**
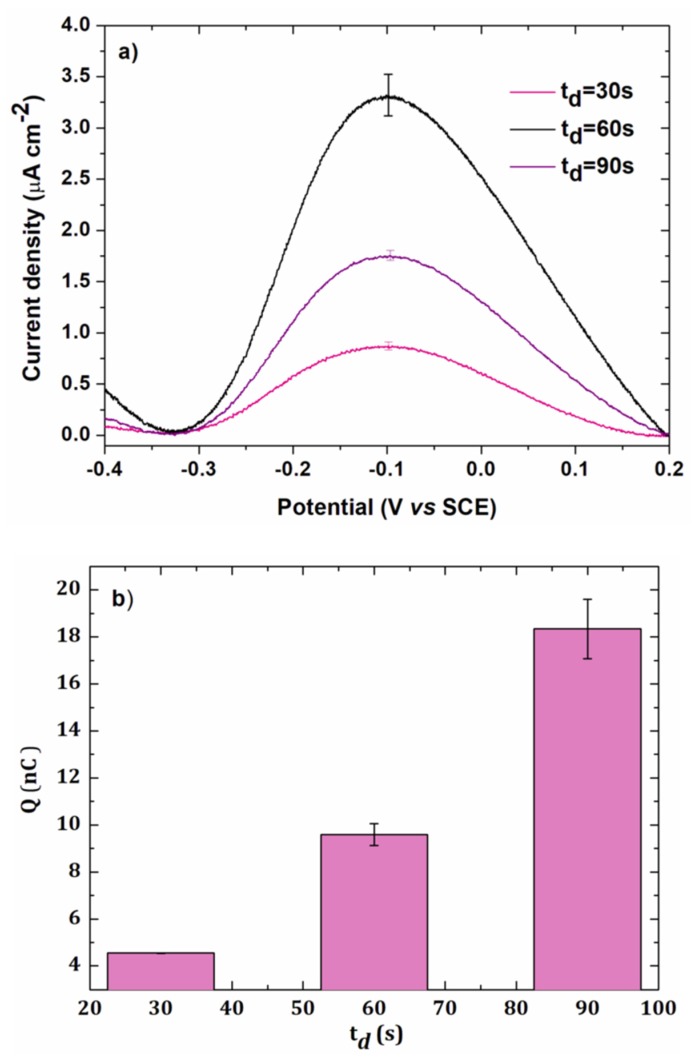
(**a**) Anodic stripping voltammetry of 5 × 10^−7^ mol L^−1^ lead chloride in 0.1 mol L^−1^ of NaCl for different deposition times t_d_ at gold electrode (0.002 cm²). Deposition potential: −0.5 V/SCE, scan rate: 100 mV s^−1^. (**b**) Charge as a function of electrodeposition time. Error bars were calculated from two repetitions.

Figure 4a shows the anodic redissolution peaks obtained in linear voltammetry with different electrodeposition times t_d_.

Figure 4b represents the total charge Q as a function of the electrodeposition time. The charge increased with t_d_ up to 90 s. A decrease was observed when higher electrodeposition times were used (120 s), probably due to a weak adherence between lead and the gold electrode surface. A time of 90 s was then chosen in further experiments as a good compromise between the analysis time and the current intensity.

The surface area occupied by lead on a flat gold surface has been estimated to be 1.6 × 10^−9^ mol cm^−2^ [31]. For a gold surface of 0.002 cm², the number of moles of lead for a monolayer is:

$$n=1.6\;\times \;10^{-9}\;\times \;0.002=3.2\;\times 10^{-12}mol$$

This corresponds to a charge of:

Q=2nF=620 nC

This value is 33 times higher than the experimental value, showing that a submonolayer was obtained after an electrodeposition step of 90 s. It confirmed the UPD electrodeposition of lead on the electrode surface.

### 3.2. Anodic Stripping Voltammetry on an Electrode Array

**Figure 5:**
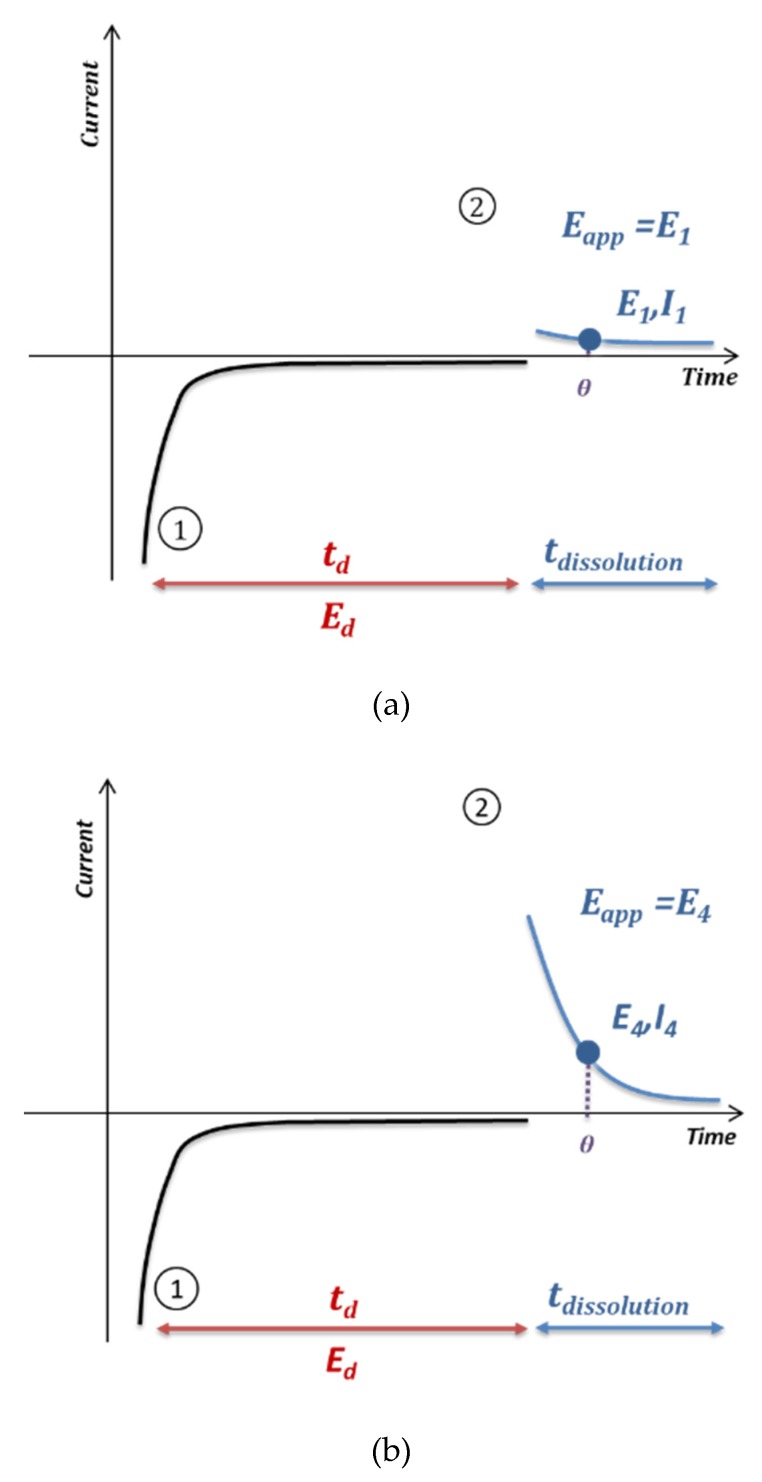

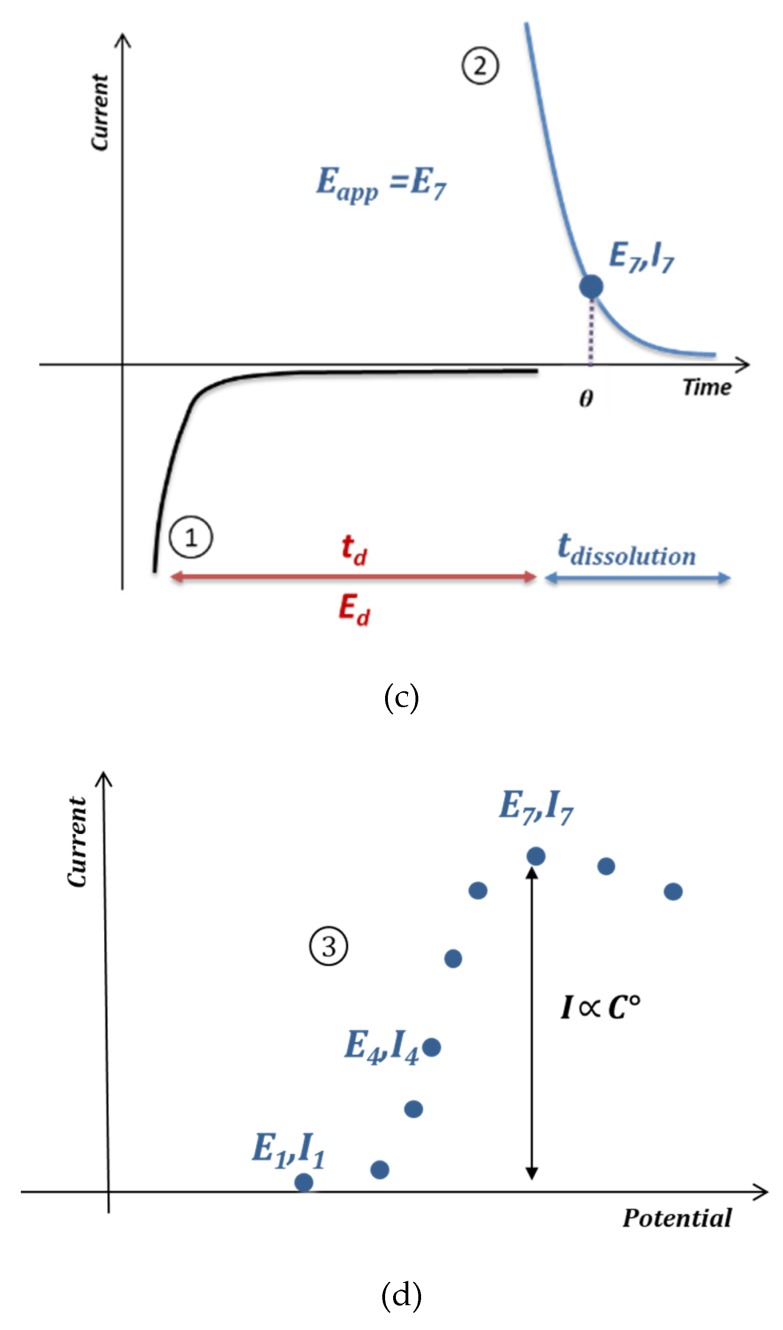
Principle of anodic stripping voltammetry on an electrode array; Electrodeposition step (1) and oxidation of species accumulated on the electrode (2) by chronoamperometry with a different potential for each working electrode number 1 (a), 4 (b), 7 (c) and sampling of the current for a defined time *θ*, leading to the I-E curve 3 (d).

Figure 5 shows the method developed to perform anodic stripping voltammetry at the electrode array. First, on each electrode of the array, an electrodeposition of lead was carried out under the conditions previously optimized on a single electrode (−0.5 V/SCE, 90 s). Then a chronoamperometry analysis was performed giving rise to the dissolution of the accumulated lead on the electrode surface. To perform the EASCV method, an increasing potential was applied on each electrode of the array. The current value was recorded for a selected sampling time θ, allowing the I-E curve to be drawn. The sampling time was selected according to several parameters such as kinetics of reaction, response time of the potentiostat and type of reaction. This curve presented a maximum value, which was proportional to the concentration of redox species in solution.

After each analysis, the electrode array was electrochemically oxidized and then washed in concentrated HNO_3_, followed by extensive rinsing with ultra-pure water.

**Figure 6:**
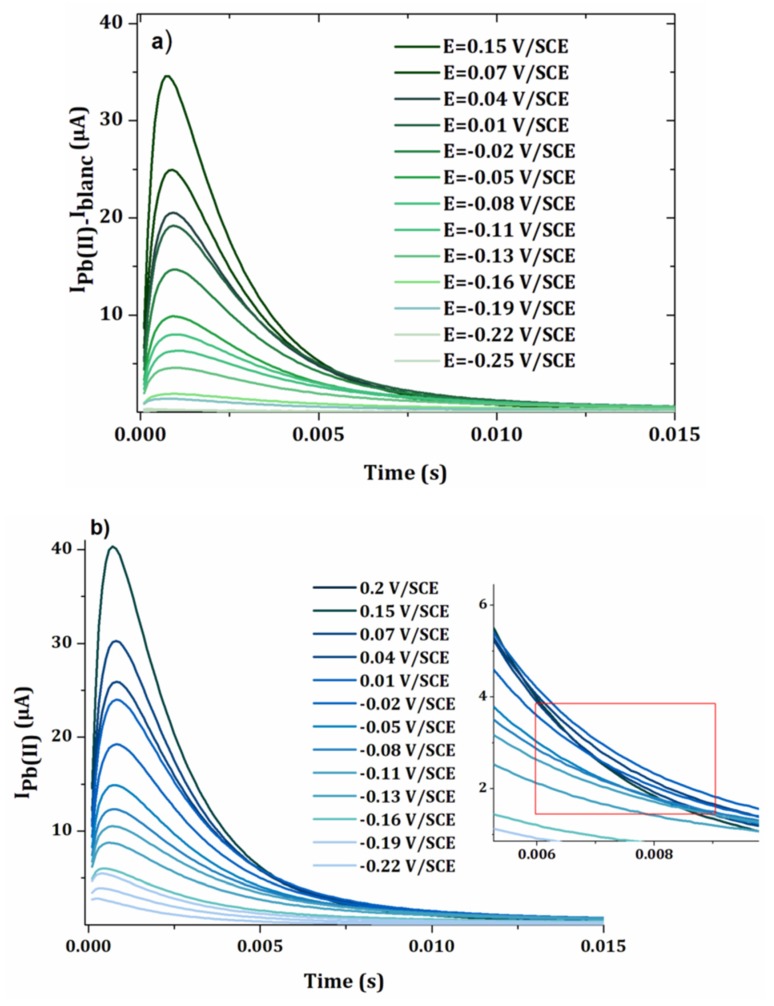
Chronoamperograms obtained by applying different potentials at the electrode array (gold electrodes 0.002 cm^2^) after an electrodeposition step at −0.5 V/SCE for 90 s of a solution of 15 µmol L^−1^ of lead chloride in 0.1 mol L^−1^ NaCl. The blank corresponds to the same analysis performed in a solution without lead. (a) Electrochemical signal of lead minus the blank (b) Electrochemical signal of lead.

A first test was carried out for a concentration of 15 μmol L^−1^ of lead chloride. Figure 6 shows the curves corresponding to the current *versus* time with and without blank subtraction. The potentials applied to the electrodes of the array were incremented by 0.03 V between −0.25 V/SCE and 0.15 V/SCE. A current peak was observed at short times, which has been previously linked to the response time of the potentiostat [22]. However, its thickness was lower than the peak previously observed for phenol analysis by EASCV, although the same potentiostat was used for both experiments. This difference in behavior shows that the sampling time is highly dependent on the equipment (potentiostat, electrodes) but also on the studied species and medium.

The variation of the current was higher in the presence of lead compared with the blank, with current values about seven times higher than in the absence of lead (Figure 6a,b).

**Figure 7:**
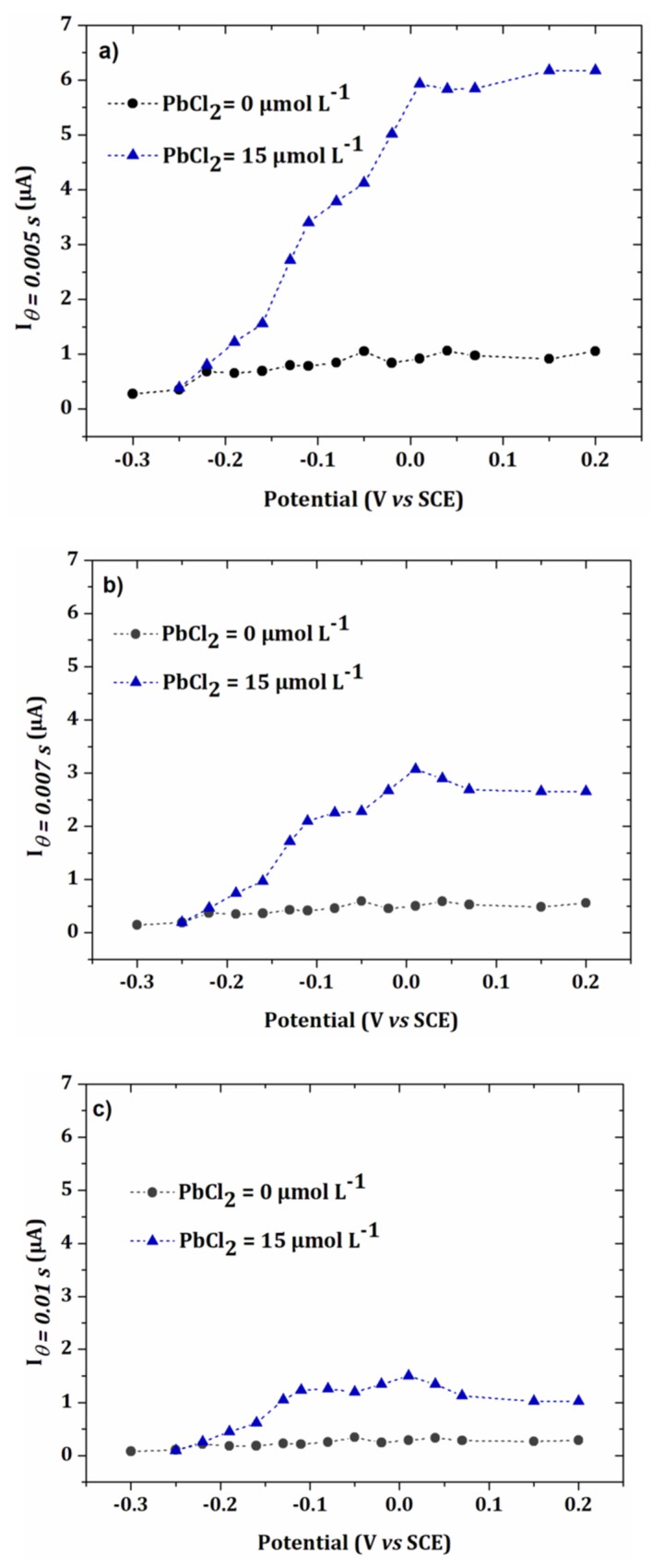
I-E curves obtained from chronoamperomety of Figure 6 for θ equal to (**a**) 0.005 s, (**b**) 0.007 s and (**c**) 0.01 s.

Figure 7a−c show the intensity-potential curves obtained from the chonoamperometry presented in Figure 6a,b for sampling times θ = 0.005 s, 0.007 s and 0.01 s. As expected, the intensity of the current decreased when the sampling time was longer. For the two highest times 0.007 s and 0.01 s, the current decreased after 0.02 V/SCE. This can be explained by the faster kinetics of the oxidation of lead at more positive potentials, leading to more rapid decrease in the current *versus* time. In Figure 6a,b, it results in a crossover of the chronoamperometry curves, for sampling times higher than 0.005 s. For the establishment of the calibration curve, a current sampling time of 0.005 s was used.

The onset potential corresponding to the oxidation of lead was around −0.2 V/SCE, which corresponded to the potential of UPD1 in Figure 2. However, the presence of two plateaus or peaks was observed, which can be explained by a difference in crystallography of the electrode material or lateral interactions between the atoms [21,28].

**Figure 8:**
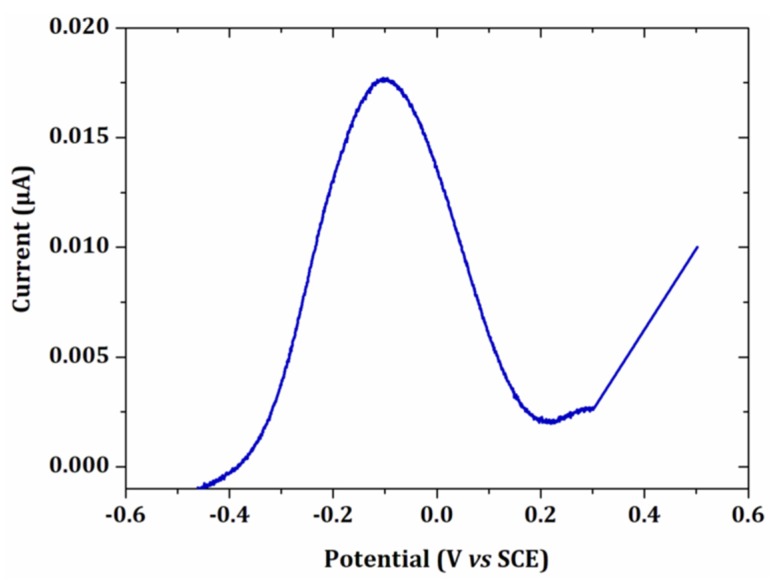
Linear voltammetry of 15 µmol L^−1^ PbCl_2_ in 0.1 mol L^−1^ NaCl after an electrodeposition at −0.5 V/SCE for 90 s. Scan rate: 100 mV s^−1^. Electrode surface: 0.002 cm².

To show the interest of the method compared to linear sweep anodic stripping voltammetry, an analysis of a lead solution was carried out under the same electrodeposition conditions and with the same electrode surface (Figure 8).

As expected, the current intensity of the peak obtained by linear voltammetry was at least 300 times lower than the maximum value obtained by EASCV. The difference was more pronounced if the sampling time was shorter. Indeed, in linear voltammetry, lead deposited on the electrode surface began to be reoxidized from −0.45 V/SCE and when the potential of the peak was reached, a high amount of lead was already oxidized. In EASCV, each electrode was independent of each other and the same initial amount of lead was present on the electrode surface when a new potential was applied. The high current intensities is an advantage of EASCV since the method does not require a Faraday cage and it is also very promising for application with ultramicroelectrodes.

### 3.3. Lead Calibration Curve Established by Coupling Anodic Stripping Voltammetry with EASCV

To simplify, the variation of the current as a function of lead concentration was studied by applying three potentials close to the maximum of current observed in Figure 7 (0.07, 0.085 and 0.1 V/SCE). Thus, an analysis of five concentrations of PbCl_2_ was performed on the same electrode array.

**Figure 9:**
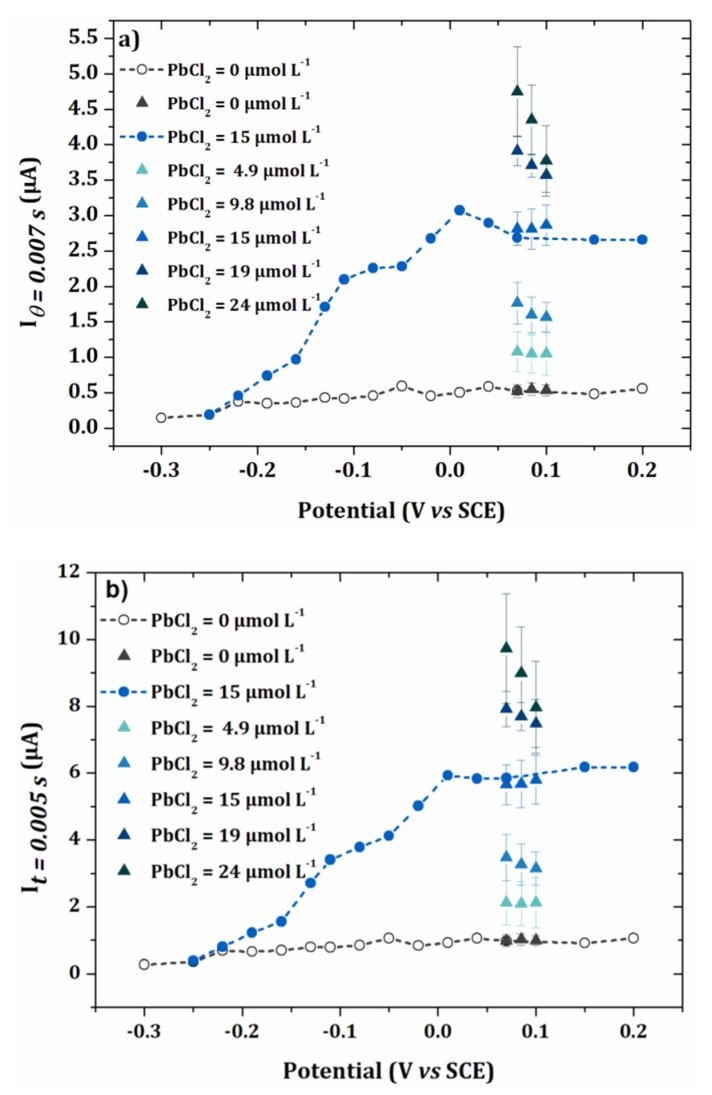
I-E curves obtained for the blank and 15 µmol L^−1^ PbCl_2_ in 0.1 mol L^−1^ NaCl. Values of current for applied potentials of 0.07 V/SCE, 0.085 V/SCE and 0.1 V/SCE for PbCl_2_ concentrations ranging from 15 µmol L^−1^ to 24 µmol L^−1^ (error bars are based on 3 repetitions) and for the blank (error bars are based on 5 repetitions). (a) θ = 0.005 s and (b) θ = 0.007 s.

This experiment was repeated three times and the average obtained is shown in Figure 9, for two different values of *θ*: 0.005 s and 0.007 s. In Figure 9a,b, the currents obtained for the three selected potentials were close to those of the I-E curves of Figure 7.

**Figure 10:**
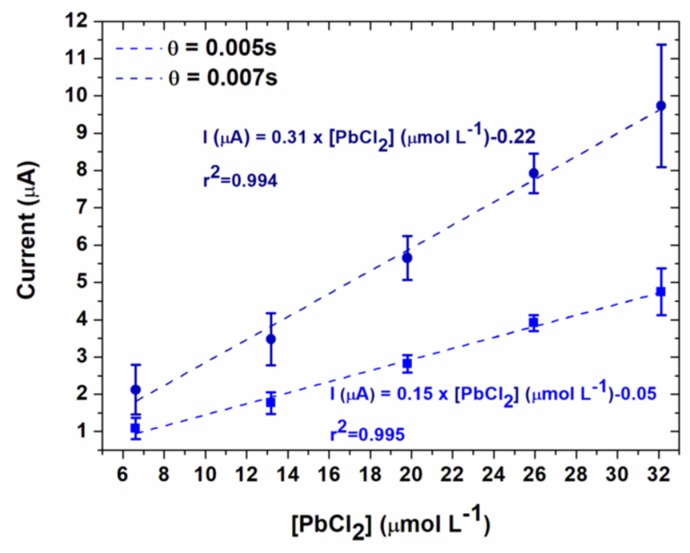
Calibration curves obtained for two sampling times, for chronoamperometry performed at 0.07 V/SCE in 0.1 mol L^−1^ NaCl (electrodeposition conditions: E_d_ = −0.5 V/SCE, t_d_ = 90 s).

For highest concentrations of lead, the current decreased when the potential increased, probably due to the higher kinetics of the oxidation reaction. Therefore, the calibration curves were plotted from the current values obtained at 0.07 V/SCE. These calibration curves for the two different sample times are given in Figure 10. The limit of quantification was determined from the following equation [32]:

St−Sb ≥ 5σ

With St the signal of the analyte at 0.07 V/SCE, Sb the mean value and σ the standard deviation of the blank calculated from five analyses at 0.07 V/SCE.

Thus, the limits of quantification were 1.39 mg L^−1^ for *θ* = 0.005 s and 1.41 mg L^−1^ for 0.007 s. These data clearly demonstrate the influence of the sampling time on the results. First, a shorter sampling time led to higher current intensities and to a higher slope of the calibration curve (Table 1). Therefore an improved accuracy was obtained on the measurement of the concentration for a given range of currents. A slightly lower limit of quantification was also found. However, the effect on the limit of quantification was not so high probably due to the high capacitive current at short sampling times.

**Figure 11:**
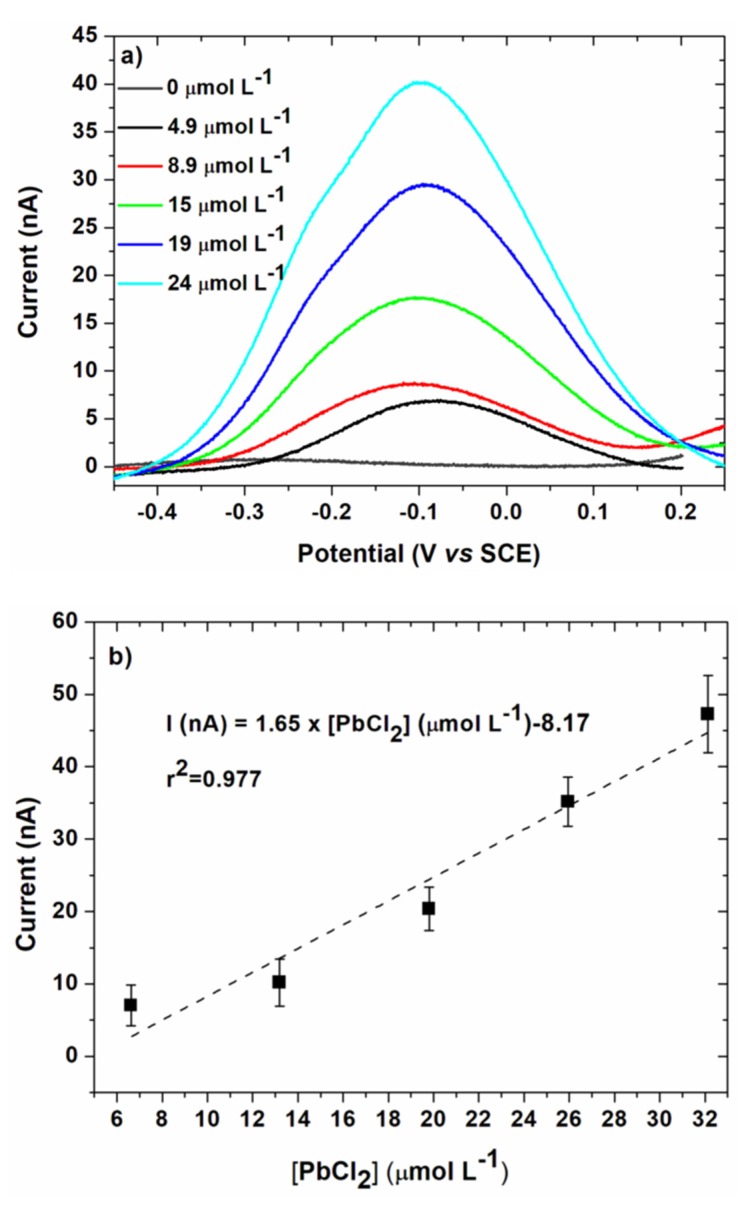
(**a**) Linear voltammetry obtained after substraction of the baseline for the same conditions as Figure 10 (100 mV s^−1^); (**b**) Corresponding calibration curve in 0.1 mol L^−1^ NaCl. Electrodeposition was performed at E_d_ = −0.5 V/SCE, t_d_ = 90 s. Error bars are based on 3 measurements.

For each concentration tested, after the electrodeposition step, a linear voltammetry was performed to compare the results with those obtained in EASCV. Results are given in Figure 11 with the corresponding calibration curve. The maximum of the anodic peak was used to plot the calibration curve to compare the data in the same unit.

A good linearity was more difficult to obtain in the studied range of concentrations, as highlighted by the regression coefficient (Table 1).

The sensitivity was significantly lower than for EASCV owing to the lower current range. Owing to this low current range, a Faraday cage was necessary to obtain a good precision of the current values. It is also interesting to note that significantly lower sensitivities were previously reported for anodic stripping voltammetry of lead performed on gold electrode (0.043 [33] and 0.016 mA mol^−1^ L [12]). However, the limit of quantification (1.03 mg L^−1^) was similar to those of EASCV. For comparison, the limit of detections LOD of sensors with gold working electrodes reported in literature are given in Table 2. LOD are significantly higher than those found in this study (1.16 mg L^−1^ in EASCV and 1.02 mg L^−1^ in linear voltammetry). It underlines that the performances of the sensor should be improved by changing the nature and size of the working electrodes of the array.

The aim of this article being the effect of coupling EASCV with an electrodeposition step on the current signal, the selectivity of the device was not studied and similar interferents already reported for anodic stripping voltammetry on gold electrode [12,13,14,33] are expected.

## 4. Conclusions

This study proposes a new analytical technique for the detection of trace metals by coupling EASCV with anodic stripping voltammetry. As a first advantage, the simplicity of the method would allow analyses on field with a portable system. Since a high electrochemical signal was expected with this method, under potential deposition of lead on the gold electrode array was used to simplify the electrochemical signal. Interestingly, the comparison of this technique with linear voltammetry showed that the maximum current intensity was 300 times higher for a same concentration. It results in a significantly higher sensitivity of the sensor given by the slope of the linear calibration curve. This result is very promising since it underlines the interest of coupling EASCV with a preconcentration step. The application of this first sensor for lead detection led to a limit of quantification of 1.16 mg L^−1^, which was similar to those of anodic stripping voltammetry on a single electrode. This value is high to measure lead in drinking water (10 μg L^−1^), but it is close to the limit of 0.5 mg L^−1^ for industrial wastewater according to the decree of 2 February 1998 [34]. An improvement of the nature and size of the working electrodes would allow the achievement of a more performant sensor. Furthermore, work is currently in progress to improve the sensitivity of the method by reduction of the capacitive current that is high at short sampling time.

## Figures and Tables

**Table 1:** Performances of the analytical methods after electrodeposition of lead at −0.5 V/SCE for 90 s on gold electrode without stirring.

Method	Sensitivity (mA mol^−1^ L)	Regression Coefficient	LOD (mg L^−1^)	LOQ (mg L^−1^)
EASCV 0.005 s	307	0.994	1.16	1.41
EASCV 0.007 s	148	0.995	1.16	1.39
Linear voltammetry	1.64	0.977	1.02	1.03

**Table 2:** Selected examples of gold working electrodes for lead detection.

Working Electrode	Electrodeposition Time	Stirring Conditions	Detection Limit (g L^−1^)	Reference
Gold screen-printed electrode	120 s	Yes	0.5	[12]
Gold-coated ultra-microelectrode	15 min	No	0.3	[13]
Gold-coated screen-printed electrode	20 min 4 min 10 min	Yes Yes No	0.1 0.6 3	[14]
Gold-coated screen-printed electrode	120 s	Yes	0.5	[33]

## References

[1] EU (1998). Directive 98/83/CE of Council of 3 November 1998 onthe quality of water intended for human consumption. Off. J. Eur. Commun..

[2] Gao C., Yu X.-Y., Xiong S.-Q., Liu J.-H., Huang X.-J. (2013). Electrochemical Detection of Arsenic(III) Completely Free from Noble Metal: Fe3O4 Microspheres-Room Temperature Ionic Liquid Composite Showing Better Performance than Gold. Anal. Chem..

[3] Nasraoui R., Floner D., Geneste F. (2009). Analytical performances of a flow electrochemical sensor for preconcentration and stripping voltammetry of metal ions. J. Electroanal. Chem..

[4] Nasraoui R., Floner D., Geneste F. (2010). Improvement in performance of a flow electrochemical sensor by using carbamoyl-arms polyazamacrocycle for the preconcentration of lead ions onto the electrode. Electrochem. Commun..

[5] Nasraoui R., Floner D., Paul-Roth C., Geneste F. (2010). Flow electroanalytical system based on cyclam-modified graphite felt electrodes for lead detection. J. Electroanal. Chem..

[6] Feier B., Fizesan I., Meriadec C., Ababou Girard S., Cristea C., Sandulescu R., Geneste F. (2015). Influence of the electrografting method on the performances of a flow electrochemical sensor using modified electrodes for trace analysis of copper (II). J. Electroanal. Chem..

[7] Feier B., Floner D., Cristea C., Bodoki E., Sandulescu R., Geneste F. (2012). Flow electrochemical analyses of zinc by stripping voltammetry on graphite felt electrode. Talanta.

[8] Feier B., Floner D., Cristea C., Sandulescu R., Geneste F. (2013). Development of a novel flow sensor for copper trace analysis by electrochemical reduction of 4-methoxybenzene diazonium salt. Electrochem. Commun..

[9] Economou A., Fielden P.R. (2003). Mercury film electrodes: developments, trends and potentialities for electroanalysis. Analyst.

[10] Bonfil J., Brand M., Kirowa-Eisner E. (1999). Determination of sub-μg l-1 concentrations of copper by anodic stripping voltammetry at the gold electrode. Anal. Chim. Acta.

[11] Bonfil Y., Brand M., Kirowa-Eisner E. (2000). Trace determination of mercury by anodic stripping voltammetry at the rotating gold electrode. Anal. Chim. Acta.

[12] Laschi S., Palchetti I., Mascini M. (2006). Gold-based screen-printed sensor for detection of trace lead. Sens. Actuators B.

[13] Wang J., Tian B. (1993). Gold ultramicroelectrodes for on-site monitoring of trace lead. Electroanalysis.

[14] Wang J., Tian B. (1993). Mercury-free disposable lead sensors based on potentiometric stripping analysis of gold-coated screen-printed electrodes. Anal. Chem..

[15] Wang J., Tian B. (1993). Screen-printed electrodes for stripping measurements of trace mercury. Anal. Chim. Acta.

[16] Duarte K., Justino C.I.L., Freitas A.C., Gomes A.M.P., Duarte A.C., Rocha-Santos T.A.P. (2015). Disposable sensors for environmental monitoring of lead, cadmium and mercury. Trends Anal. Chem..

[17] Jothimuthu P., Wilson Robert A., Herren J., Haynes Erin N., Heineman William R., Papautsky I. (2011). Lab-on-a-chip sensor for detection of highly electronegative heavy metals by anodic stripping voltammetry. Biomed. Microdevices.

[18] Bonfil Y., Brand M., Kirowa-Eisner E. (2002). Characteristics of subtractive anodic stripping voltammetry of Pb and Cd at silver and gold electrodes. Anal. Chim. Acta.

[19] Bonfil Y., Brand M., Kirowa-Eisner E. (2000). Determination of mercury and copper in waste water by anodic-stripping voltammetry at the gold electrode. Rev. Anal. Chem..

[20] Bonfil Y., Kirowa-Eisner E. (2002). Determination of nanomolar concentrations of lead and cadmium by anodic-stripping voltammetry at the silver electrode. Anal. Chim. Acta.

[21] Herzog G., Arrigan D.W.M. (2005). Determination of trace metals by underpotential deposition-stripping voltammetry at solid electrodes. Trends Anal. Chem..

[22] Mazerie I., Didier P., Razan F., Hapiot P., Coulon N., Girard A., de Sagazan O., Floner D., Geneste F. (2018). A General Approach Based on Sampled-Current Voltammetry for Minimizing Electrode Fouling in Electroanalytical Detection. ChemElectroChem.

[23] Mignard L., Denoual M., Lavastre O., Floner D., Geneste F. (2013). Sampled voltammetry on an electrode array for the renewal of the electrode surface and the analytical solution during the analysis. J. Electroanal. Chem..

[24] Mazerie I. (2016). Développement de capteurs électrochimiques basés sur de la voltammétrie par échantillonnage de courant sur réseau d’électrodes. Ph.D. Thesis.

[25] Engelsmann K., Lorenz W.J., Schmidt E. (1980). Underpotential deposition of lead on polycrystalline and single-crystal gold surfaces. Part II. Kinetics. J. Electroanal. Chem..

[26] Engelsmann K., Lorenz W.J., Schmidt E. (1980). Underpotential deposition of lead on polycrystalline and single-crystal gold surfaces. Part I. Thermodynamics. J. Electroanal. Chem..

[27] Hamelin A., Katayama A., Picq G., Vennereau P. (1980). Surface characterization by underpotential deposition: lead on gold surfaces. J. Electroanal. Chem..

[28] Kirowa-Eisner E., Bonfil Y., Tzur D., Gileadi E. (2003). Thermodynamics and kinetics of upd of lead on polycrystalline silver and gold. J. Electroanal. Chem..

[29] Vicente V.A., Bruckenstein S. (1973). Rotating ring-disk electrode study of the adsorption of lead on gold in 0.5M potassium chloride. Anal. Chem..

[30] Koper M.T.M. (2003). Stripping voltammetry and chronoamperometry of an adsorbed species with repulsive lateral interactions. Z. Phys. Chem..

[31] Melroy O., Kanazawa K., Gordon J.G., Buttry D. (1986). Direct determination of the mass of an underpotentially deposited monolayer of lead on gold. Langmuir.

[32] MacDougall D., Crummett W.B. (1980). Guidelines for data acquisition and data quality evaluation in environmental chemistry. Anal. Chem..

[33] Mandil A., Idrissi L., Amine A. (2010). Stripping voltammetric determination of mercury(II) and lead(II) using screen-printed electrodes modified with gold films, and metal ion preconcentration with thiol-modified magnetic particles. Microchim. Acta.

[34] World Health Organization (2011). Adverse health effects of heavy metals in children. Children’s Health and the Environment WHO Training Package for the Health Sector World.

